# Longitudinal Study of Viral Diversity Associated with Mosquito Species Circulating in Cambodia

**DOI:** 10.3390/v15091831

**Published:** 2023-08-29

**Authors:** Souand Mohamed Ali, Antsa Rakotonirina, Kimly Heng, Elise Jacquemet, Stevenn Volant, Sarah Temmam, Sebastien Boyer, Marc Eloit

**Affiliations:** 1 Pathogen Discovery Laboratory, Institut Pasteur, Université de Paris, 75015 Paris, France; souand.mohamed-ali@pasteur.fr (S.M.A.); sarah.temmam@pasteur.fr (S.T.); 2 Medical and Veterinary Entomology Unit, Institut Pasteur du Cambodge, Phnom Penh 12201, Cambodia; arakotonirina@pasteur-kh.org (A.R.); sboyer@pasteur-kh.org (S.B.); 3 Bioinformatics and Biostatistics Hub, Institut Pasteur, Université Paris Cité, 75015 Paris, Francestevenn.volant@pasteur.fr (S.V.); 4 Ecology and Emergence of Arthropod-Borne Diseases, Institut Pasteur, 75015 Paris, France; 5 Ecole Nationale Vétérinaire d’Alfort, University of Paris-Est, 94704 Maisons-Alfort, France

**Keywords:** virus discovery, mosquito, mosquito virome, comparative metagenomic, Cambodia

## Abstract

Arthropod-borne viruses (arboviruses) pose a significant global health threat and are primarily transmitted by mosquitoes. In Cambodia, there are currently 290 recorded mosquito species, with at least 17 of them considered potential vectors of arboviruses to humans. Effective surveillance of virome profiles in mosquitoes from Cambodia is vital, as it could help prevent and control arbovirus diseases in a country where epidemics occur frequently. The objective of this study was to identify and characterize the viral diversity in mosquitoes collected during a one-year longitudinal study conducted in various habitats across Cambodia. For this purpose, we used a metatranscriptomics approach and detected the presence of chikungunya virus in the collected mosquitoes. Additionally, we identified viruses categorized into 26 taxa, including those known to harbor arboviruses such as *Flaviviridae* and *Orthomyxoviridae*, along with a group of viruses not yet taxonomically identified and provisionally named “unclassified viruses”. Interestingly, the taxa detected varied in abundance and composition depending on the mosquito genus, with no significant influence of the collection season. Furthermore, most of the identified viruses were either closely related to viruses found exclusively in insects or represented new viruses belonging to the *Rhabdoviridae* and *Birnaviridae* families. The transmission capabilities of these novel viruses to vertebrates remain unknown.

## 1. Introduction

Mosquitoes have consistently been identified as the primary vectors of arthropod-borne viruses (arboviruses) in numerous studies and epidemiological investigations [1,2,3,4]. Over 300 mosquito species, mainly belonging to the *Aedes* and *Culex* genera [5,6], are capable of transmitting arboviruses.

Arboviruses pose an increasing public health threat, imposing significant social and economic burdens in various countries globally. There are over 500 known circulating arbovirus strains, approximately 100 of which can cause harm to both humans and animals [7]. These include well-known viruses such as Yellow fever virus (YFV), Zika virus (ZIKV), Dengue virus (DENV), and Chikungunya virus (CHIKV) [8,9,10], as well as Japanese encephalitis virus (JEV) and West Nile virus (WNV), carried by *Aedes* and *Culex* mosquitoes [11,12].

In addition to arboviruses, certain mosquito species also harbor a class of viruses known as insect-specific viruses (ISVs). ISVs have a limited host range restricted to insects and cannot infect vertebrates or replicate in vertebrate cell lines [13,14,15,16]. The first identified ISV was the Cell-Fusing Agent Virus (CFAV), a flavivirus that infects *Ae. aegypti* cell lines and exhibits a significant cytopathic effect in *Ae. albopictus* cell lines. BHK-21 isolated from the kidney of a golden hamster, Vero, derived from the kidney of an African green monkey, and BK, known to be a subline of the widely prevalent keratin-forming tumor cell line HeLa [17]. The impact of ISVs on the transmission dynamics of arboviruses by mosquitoes has been the subject of investigation in recent years. Several studies have provided evidence that ISVs play a significant role in modulating the ability of mosquitoes to transmit arboviruses. For instance, the presence of ISVs in mosquito saliva has been found to competitively limit the replication of arboviruses by a mechanism involving resource competition within the cellular environment, thereby blocking the transmission process [18]. Additionally, ISV infections in mosquitoes have been shown to trigger an immune response, leading to the activation of antiviral mechanisms. This immune response plays a pivotal role in restricting arboviral replication within mosquitoes, consequently limiting their transmission to human or vertebrate hosts [19].

Moreover, recent years have seen the discovery of less characterized viruses called mosquito-associated viruses (MAVs) [20,21]. Unlike ISVs, MAVs have not been the subject of experimental studies to assess their ability to replicate in vertebrate hosts, their pathogenicity, or their impact on arbovirus replication within mosquitoes. The only known information about MAVs is that they are solely detected in mosquitoes.

The spread of mosquito vectors is facilitated by factors such as climate change, mosquito adaptability, rapid urbanization, and increased international travel and trade, leading to the expansion of regions affected by arboviruses [22]. One of the countries significantly impacted by climatic change and facing the burden of arbovirus diseases is Cambodia, located in Southeast Asia. The country faces challenges such as floods, which can influence mosquito breeding, thereby increasing the risk of arbovirus emergence [23]. The national sentinel surveillance system has reported a high annual average of 103 cases of dengue fever per 10,000 population since 2000. The case fatality rate for dengue fever ranges from 1% to 2%, placing Cambodia among the most affected countries in Southeast Asia [24]. Japanese encephalitis is also endemic and a leading cause of acute encephalitis, particularly in children [25,26,27]. Zika virus was also infrequently reported from 2007 to 2016 [28]. Chikungunya re-emerged in 2020 and led to a nationwide outbreak [29].

Additionaly, Cambodia harbors a diverse mosquito population, comprising more than 290 species from 20 genera, with 43 identified as vectors of pathogens [30]. This diversity contributes to the complexity of controlling arbovirus transmission in the country. To mitigate the risk of arbovirus emergence, comprehensive vector control measures, strengthened surveillance systems, and increased public awareness on arbovirus prevention and control are crucial.

To better measure the virus burden in mosquitoes, it is necessary to adopt broad-range methods capable of also detecting unknown viruses. In recent years, metagenomic analysis of field-collected mosquitoes using advanced next-generation sequencing (NGS) technology has emerged as a valuable tool. This approach allows for the detection of a wide range of novel or unexpected viruses [31,32]. In this study, using this tool, we have contributed to the understanding of arbovirus disease epidemiology and circulation in Cambodia by conducting a one-year longitudinal study in different biotopes.

## 2. Materials and Methods

### 2.1. Mosquito Collection and Identification

This study was approved by Cambodia authorities, with an authorization letter from the Ministry of Environment issued in November 2019 (permit no 144). Mosquito sampling was carried out in eight communes located in Kampong Thom Province. A total of six environmental sites were selected (Figure S1). Eleven missions were conducted between January 2021 and December 2021. For each mission, each site was investigated during three consecutive days using two BG-1 Sentinel™ Mosquito Traps, 7.5–12 Volt baited with BG-Lure^®^ (BioQuip, Rancho Dominguez, CA, USA) installed both inside and outside houses. Dry ice was placed in a dry ice dispenser next to each trap. Each trapping location was visited every day to remove the collected insects. Caught mosquitoes were subsequently killed using carbon dioxide and further morphologically identified using available identification keys [33,34,35,36]. Mosquitoes were stored immediately at 4 °C at the field until returning to the laboratory, where they were stored at −80 °C until further experiments.

### 2.2. Preparation of Metatranscriptomics Libraries

#### 2.2.1. Mosquito Pooling and RNA Extraction

Collected mosquitoes were pooled per species, season, and collection location (indoor/outdoor) to a total of 10 mosquitoes maximum per minipool, and homogenized with 500 µL of PBS using a MagnaLyser version 1.1 (Roche, Mannheim, Germany) at 6000 rpm for 1 min. Crushing material was centrifuged for 2 min at 12,000× *g* and 4 °C, then 167 µL of supernatant was transferred individually to 835 µL of RNA later solution (Invitrogen). The mixture was incubated overnight at 4 °C and stored at −80 °C until shipment to Institut Pasteur in Paris. According to the mosquito species season and collection location (indoor/outdoor), minipools were combined to form large pools that contained a maximum of 100 mosquitoes per pool (Table S1). A total of 6646 mosquitoes were selected and subsequently distributed across 103 large pools. Overall, total RNA was extracted from the 103 large pools of mosquitoes in a Biosafety Level 3 (BSL-3) laboratory using the Maxwell RSC simply RNA tissue kit (Promega, Madison, WI, USA), according to the manufacturer’s instructions. RNA extracts were quantified with the Qubit RNA High sensitivity assay (Invitrogen, Waltham, MA, USA) and analyzed using an Agilent BioAnalyzer RNA pico chip (Agilent, Waldbronn, Germany).

#### 2.2.2. NGS Library Preparation and Sequencing

Sequencing libraries of the 103 large pools were prepared using the SMARTer Stranded Total RNA-seq kit v3-Pico input mammalian kit (Takara Bio, San Jose, CA, USA). The quantity of RNA input, the duration of heat fragmentation, and the final amplification were adapted according to each sample RNA profile. Quantification and quality controls of the libraries were verified by the Qubit DNA High sensitivity assay (Invitrogen) and the Bioanalyzer DNA High Sensitivity chips (Agilent, Waldbronn, Germany), respectively. Sequencing was carried out on the Illumina NovaSeq or NextSeq 2000 devices in a paired-ends 2 × 150 bp or 2 × 100 bp format, respectively, to achieve approximately 50 million reads for each library (Table S2).

### 2.3. Virus Assignment

Raw reads were processed with an in-house bioinformatics pipeline (Microseek, Institut Pasteur, Paris, France) that allowed for quality check followed by read trimming and normalization [37]. Trimmed reads were de novo assembled and translated into protein sequences using an in-house translation tool comprised in Microseek. A BLAST-based similarity search was then performed for all contigs and singletons against the comprehensive and curated protein Reference Viral database (RVDB-prot) [38] followed by a BlastP-based verification of the accuracy of the viral taxonomic assignation against the whole protein NCBI/nr database. A final BLASTN-based verification was performed against NCBI/nt to confirm that no better hit was obtained with non-coding sequences present in NCBI/nt. The quantification of abundance of each viral taxon was estimated by summing the length (in nucleotides) of all sequences (contigs and singletons) associated to this taxon instead of summing the raw number of sequences, in order to take into account the length and depth of long viral contigs.

### 2.4. Phylogenetic Analyses

To determine the evolutionary history of newly discovered viruses, amino acid sequences of the complete viral polymerase or complete polyprotein of each virus were compared with the sequences of the same type of proteins recovered from the NCBI database belonging to the same family. Within each family, sequences were aligned by using the E-INS-I algorithm in MAFFT (version 7) [39]. Ambiguously aligned regions were subsequently removed using BMGE [40]. Phylogenetic trees were constructed with PhyML (version 3.1) [41], employing LG as the evolutionary and substitution model and Subtree Pruning and Regrafting (SPR) as the tree topology. Approximate Bayes parameter implemented in PhyML was used for branch support statiscal test. Phylogenetic trees were visualized with the Interactive Tree Of Life tool (iTOL version 6) [42].

### 2.5. Statistical Analyses

Principal Coordinates Analysis (PCoA) was conducted to explore differences in viral abundances between species, season, and collection location (Indoor/Outdoor). PCoA was based on the Bray−Curtis dissimilarity distance and computed using R software (v4.2.1) and ade4 package [43]. Differential abundance analysis was performed using SHAMAN with default parameters [44]. Abundance count data were normalized following the normalization method provided in the DESeq2 R package (v1.6.3) [45]. To identify differentially abundant viral genera across mosquito genera, a generalized linear model (GLM) was used. The GLM included mosquito genera as the main effect. Covariates season, collection location, and sequencing batch were included to take into account their potential effects. The resulting *p*-values were adjusted using the Benjamin and Hochberg procedure to account for multiple comparisons. Associated figures were generated with ggplot2 package.

## 3. Results

### 3.1. Mosquito Diversity and Abundance

The year 2021, during which the mosquitoes were collected, covered the two main bioclimatic seasons: the dry season from January to March and December, and the rainy season lasting from April to November.

The collected mosquitoes were classified into three genera: *Culex*, *Aedes*, and *Anopheles*, encompassing nine species: *Ae. aegypti*, *Ae. albopictus*, *An. indefinitus*, *An. vagus*, *Cx. brevipalpis*, *Cx. gelidus*, *Cx. quinquefasciatus*, *Cx. tritaeniorhyncus*, and *Cx. vishnui* group.

Among these species, the most abundant during the dry season was *Cx. quinquefasciatus*, accounting for 54% of all captured mosquitoes, followed by *Cx. vishnui* group (21%) and *Ae. aegypti* (12%). Conversely, in the rainy season, *Ae. aegypti* was the most frequently captured species (31%), followed by *Cx. vishnui* group (24%) and *Cx. quinquefasciatus* (23%) (Figure S2).

### 3.2. Overview of the Virome Composition among Mosquito Genera

**Figure 1:**
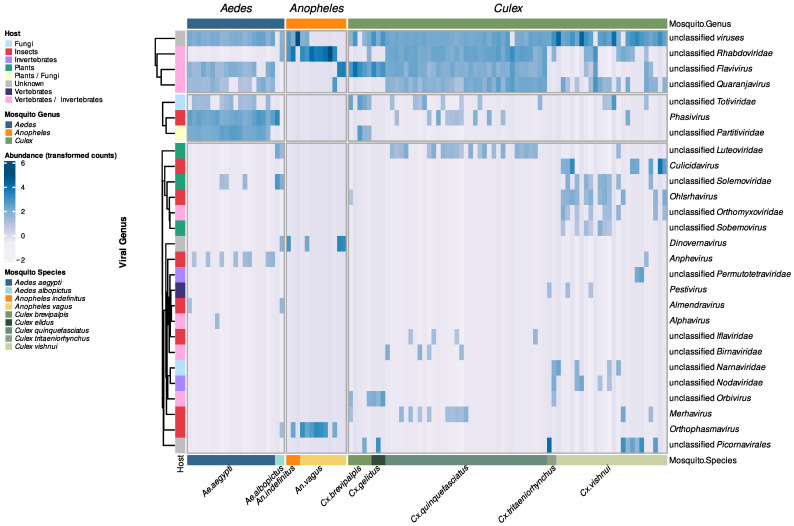
Normalized abundance of viral species in mosquito pools. The heatmap shows the viral abundances across mosquito genera and species. The normalized counts were transformed using variance stabilizing transformation (VST) and are displayed on a logarithmic scale, ranging from white (indicating low abundance) to dark blue (indicating high abundance). The viral genera (on the right) were clustered based on their relative abundances, and the corresponding hosts are shown on the left.

To visualize the composition of the virome across mosquito genera and species, we generated a heatmap representing the normalized viral abundance (Figure 1).

We identified a total of 26 taxa, including a group of viruses labeled as “unclassified viruses” by the International Committee on Taxonomy of Viruses (ICTV). The heatmap included mixed family groups and genus groups, as taxonomic assignations were based on Last Common Ancestor (LCA) information provided by Microseek. In certain cases, the LCA corresponded to a specific viral species or genus, resulting in classification at that level. In other instances, LCA was only identified at the family level, and thus the classification was maintained at that level.

The majority of the identified viruses belonged to taxa known to specifically infect insects, such as *Merhavirus* and *Ohlsrhavirus* (*Rhabdoviridae*), and *Phasivirus* (*Phenuiviridae*). Additionally, we found taxa associated with genera known to harbor arboviruses, such as *Flavivirus* (*Flaviviridae*), *Quaranjavirus* (*Orthomyxoviridae*), and *Alphavirus* (*Togaviridae*). The remaining viral sequences were assigned to taxa known to infect mostly plants and fungi (Figure 1).

**Figure 2:**
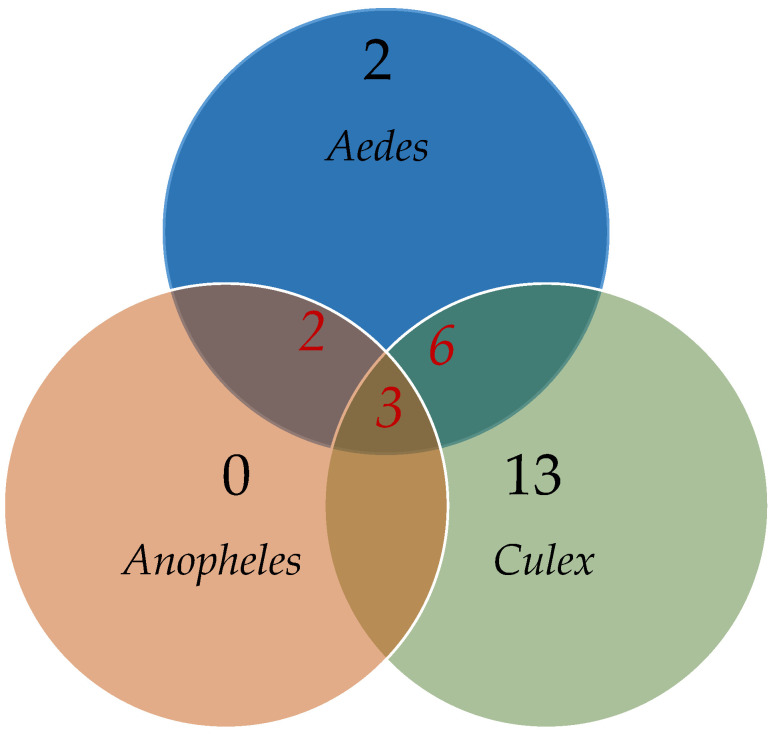
Venn diagram illustrating the number of identified taxa for each mosquito genus, with each genus represented by a circle. The numbers in the overlapping areas indicate the shared taxa between the concerned mosquito genera, while the numbers in the non-overlapping areas represent the taxa uniquely identified within each genus.

Two viral taxa, *Dinovernavirus* and *Orthophasmavirus*, were only identified in *Aedes* and *Anopheles* mosquitoes. Six viral taxa were shared exclusively between *Aedes* and *Culex* mosquitoes, including unclassified *Totiviridae* and unclassified *Partitiviridae*. Additionally, three viral taxa, namely unclassified *Rhabdoviridae*, unclassified *Flaviviridae*, and unclassified *Quaranjavirus*, were identified in all three mosquito genera. Furthermore, some taxa were exclusively found in one mosquito genus and absent in others. For instance, two taxa (*Alphavirus* and *Almendravirus*) were solely found in *Aedes* mosquitoes, while thirteen taxa (including *Culicidavirus* and *Pestivirus*) were only observed in *Culex* mosquitoes. Notably, no taxa were solely identified in *Anopheles* mosquitoes (Figure 2 and Figure S3).

### 3.3. Comparing Virome Diversity and Abundance across Mosquito Genera, Season, and Collection Location

To assess variations in viral abundance among samples, we performed a Principal Coordinates Analysis (PCoA) on 87 out of the 103 libraries. Sixteen samples were excluded from the statistical analysis due to inadequate representation, resulting in an insufficient number for statistical comparisons.

**Figure 3:**
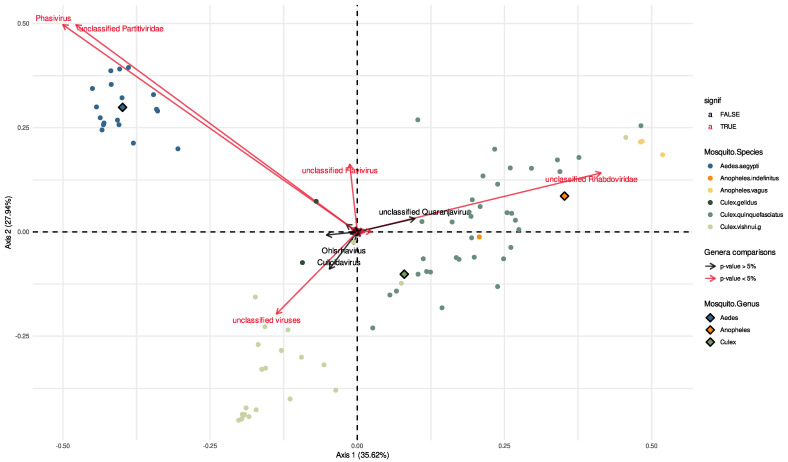
Statistical analyses comparing viral abundances among mosquito genera. Principal Coordinates Analysis was conducted on normalized relative abundances. The first two axes capture 63% of the variability within the data and highlight differences in viral composition across mosquito genera. The arrows show the direction of gradients of abundances for each viral genera and their length is proportional to the covariance between the mosquito genera and computed PcoA axis. Viral genera that showed significant differences through differential analysis are highlighted in red.

The results revealed clear differences in the virome composition and viral abundances among the mosquito genera, with three separate groups representing each mosquito genus. Furthermore, within the *Culex* genus, we encountered a substantial number of mosquito species, specifically *Cx. Vishnui* group and *Cx. Quinquefasciatus*. Notably, it appears that there was a clear division at the species level, with these two species seemingly grouped into two distinct categories (Figure 3).

However, no significant differences were observed for other covariates, namely the season and the collection location (indoor/outdoor; Figure S4).

**Figure 4:**
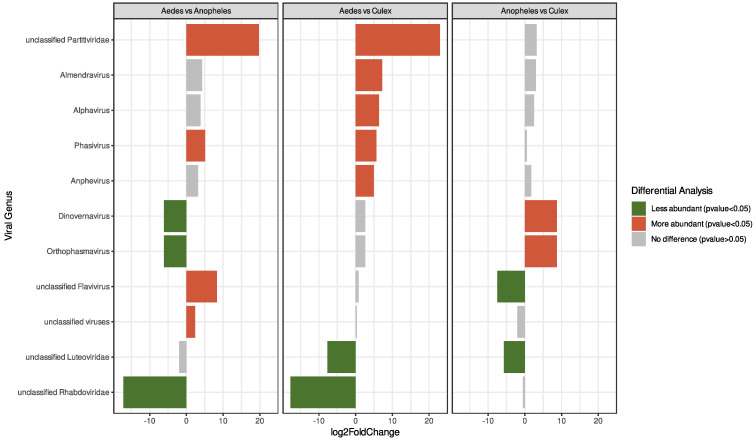
Differential analysis was conducted to identify variations in viral abundance among mosquito genera through pairwise comparisons. The fold change (log2 scale) computed during the differential analysis is displayed for significant comparisons.

To compare viral abundance among mosquito genera more effectively, we conducted a differential analysis. This allowed us to identify viral groups with varying abundance levels through pairwise comparisons, and we quantified the differences using the Fold Change criteria. Among the findings, it was observed that the unclassified *Partitiviridae* family exhibited the highest abundance in *Aedes* mosquitoes, while the *Dinovernavirus* genus was most abundant in Anopheles mosquitoes. On the other hand, the abundance of the unclassified *Rhabdoviridae* appeared to be similar between *Anopheles* and *Culex* mosquitoes (Figure 4). The comprehensive results, including the Fold Change and adjusted *p*-values for all viral groups, are also reported (Table S3).

### 3.4. Genetic Characterization of Relevant Viruses

To uncover potential arboviruses responsible for mild infections in humans, we focused on taxa of viruses known to infect both vertebrates and invertebrates. Subsequently, we conducted a comprehensive phylogenetic analysis, which enabled us to identify and classify the viral species present within these specific groups.

#### 3.4.1. Togaviridae

The family *Togaviridae* includes two genera, *Alphavirus* and *Rubivirus*. The *Rubivirus* genus contains a single virus that causes mild diseases in children, and it is transmitted by the respiratory route. The *Alphavirus* genus contains a large number of viruses, many of which are arboviruses and cause human diseases. The most common symptoms are fever, encephalitis, and rashes.

**Figure 5:**
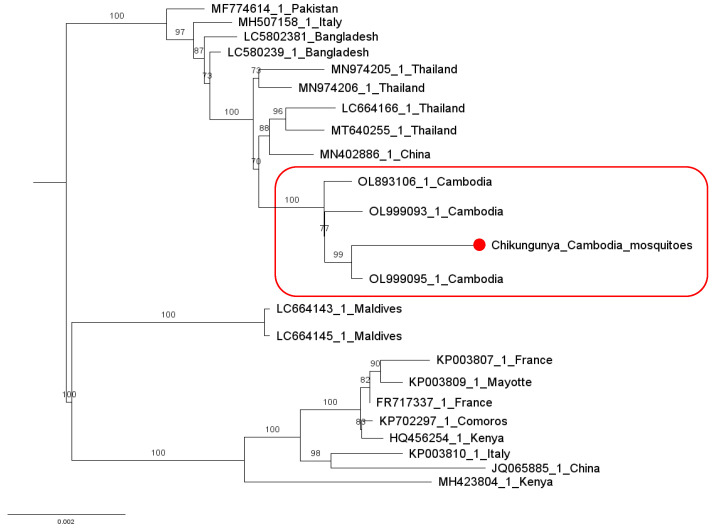
Phylogenetic analysis of Chikungunya virus strains identified in mosquitoes in Cambodia, in relation to other strains belonging to the Indian Ocean lineage. The phylogenetic tree is constructed based on the complete genomes of nucleotide sequences. The branch highlighted in red represents sequences originating from Cambodia. The sequence identified in this study is represented by a solid red circle. The scale bar indicates the number of nucleotide substitutions per site.

Within this family, we identified viral sequences assigned to Chikungunya virus (CHIKV) belonging to the *Alphavirus* genus. CHIKV was detected in one *Ae. Aegypti* pool collected in the rainy season (Table 1). The consensus sequence showed 90% genome coverage, and an overall 99.9% amino-acid identity compared with a CHIKV strain identified in Cambodia from a human serum in 2021 (OL999095). The CHIKV sequence clustered in a phylogenetic clade comprising other CHIKV strains originating from Cambodia and isolated from the serum of patients in Cambodia (Figure 5).

#### 3.4.2. Flaviviridae

The family *Flaviviridae* contains four genera approved by the ICTV: *Flavivirus*, *Hepacivirus*, *Pegivirus*, and *Pestivirus*. A new group of segmented Flaviviridae-related viruses, named “Jingmenvirus group”, has been identified and described in the literature [46,47]. Most members of this family are important human and veterinary pathogens such as Yellow fever virus, Dengue virus, and West Nile virus.

Within this family, viral sequences identified in this study were assigned to three viruses belonging to the *Flavivirus* genus (Table 1). The cell fusing agent virus (CFAV) was detected only in *Ae. Aegypti* pools. Complete coding genome sequences were obtained and showed 99% of amino-acid identity with the LR694078 strain identified from *Ae. Aegypti* collected in Cambodia in 2015. The Culex flavivirus (CxFV) was identified in pools of *Culex* and *Anopheles* mosquitoes. Complete coding genome sequences were obtained, revealing amino acid identities of 71% for *Anopheles* mosquitoes and one pool of *Cx. Brevipalpis* mosquitoes, and 99% for the other *Culex* mosquitoes when compared with the BBQ04787 and HQ678513.1 strains, which were collected from *Culex* mosquitoes in Brazil in 2017. The Quang Binh virus (QBV) was detected in *Cx. Gelidus* pools. The consensus sequences showed coverage and amino acid identity of 99% to 100%, compared with the NC_012671 strain from *Culex* mosquitoes collected in Vietnam in 2002. It is important to note that, for each virus, the identified sequences from different mosquitoes collected in different seasons shared more than 99% identity of nucleotides.

**Figure 6:**
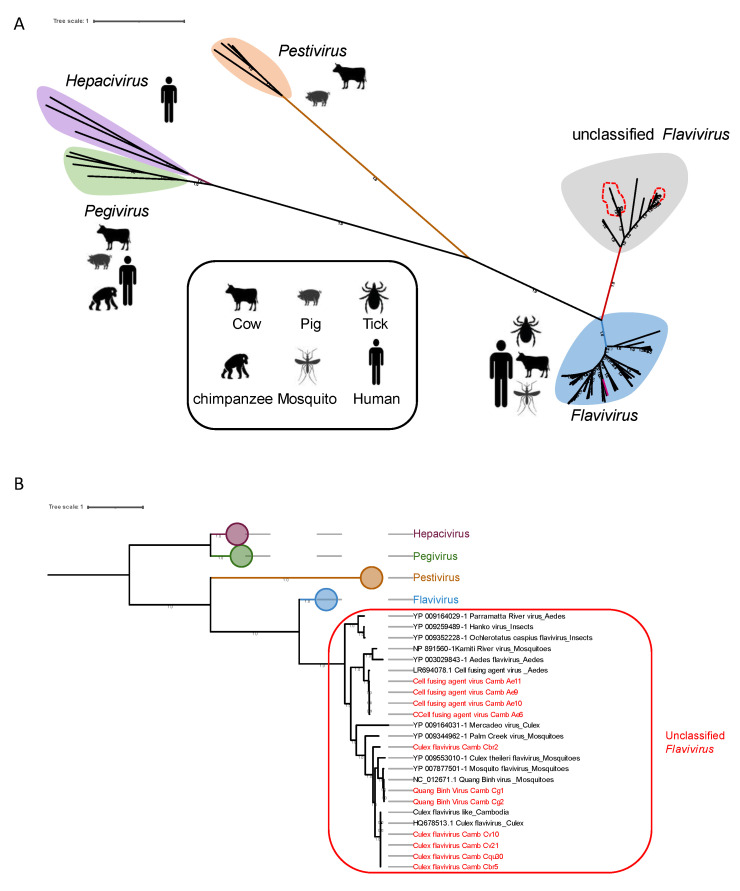
Phylogenetic analysis of *Flaviviridae* viruses identified in this study and their relationship with other family members. The construction of the phylogenetic tree was based on the complete polyprotein sequence. The labeled branches represent various known virus genera belonging to the *Flaviviridae* family, as well as sequences belonging to the unclassified *Flavivirus* group. The scale bar indicates the number of amino-acid substitutions per site. (**A**) Division of viruses according to genera within the *Flaviviridae* family. The sequences identified in this study are surrounded in red. (**B**) Enlarged view of unclassified Flaviviruses. These viruses are represented by the branch encircled in red. The sequences identified in this study are written in red.

While the *Flavivirus* genus includes a number of arboviruses, certain members within this genus are classified as ISVs including CFAV, CxFV, and QBV. The phylogenetic analysis revealed that these viruses cluster together with MAVs within a distinct clade within the *Flavivirus* genus. The ICTV designates this clade as “unclassified *Flavivirus*” (Figure 6A,B). Concerning the virus identified in Anopheles, we did not obtain an entire genome, unlike the viruses identified in Culex mosquitoes. The largest contig was 2198 amino acids long. Consequently, we did not include it in our phylogenetic analyses.

#### 3.4.3. Rhabdoviridae

The family *Rhabdoviridae* includes three subfamilies, 45 genera, and 275 virus species. Certain genera such as *Hapavirus* and *Ephemerovirus* contain arboviruses. The *Vesiculovirus* and *Lyssavirus* genera contain viruses that are pathogens for humans.

Complete coding genome sequences assigned to Guadeloupe Culex rhabdovirus were found in different *Culex* mosquitoes (Table 1). They showed 99% amino-acid identity compared with the strain MN013393 discovered in *Cx. quinquefasciatus* in Guadeloupe.

**Figure 7:**
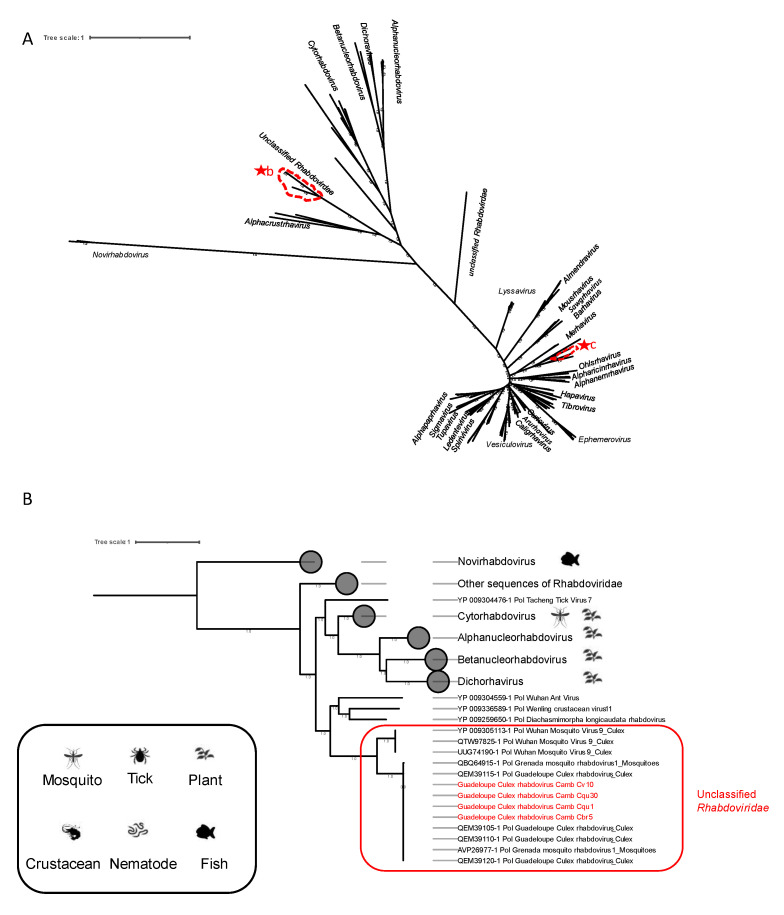

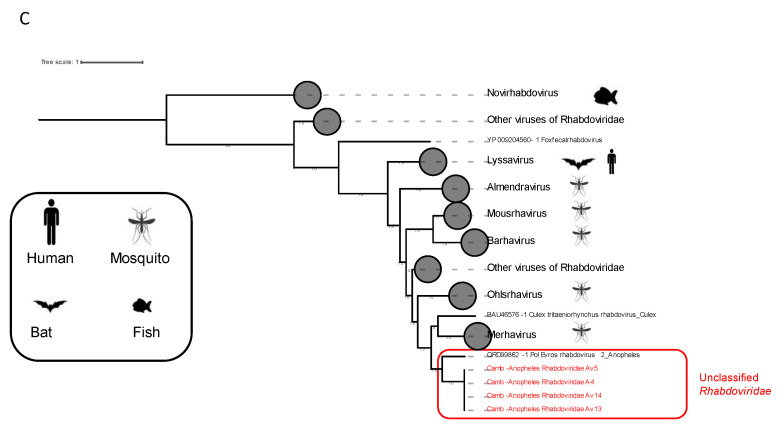
The phylogenetic analysis included viruses from the *Rhabdoviridae* family found in this study, as well as other viruses within the same family. The phylogenetic tree was constructed based on the RdRp amino acid sequence. The labeled branches represent various known virus genera belonging to the *Rhabdoviridae* family, as well as sequences belonging to the unclassified *Rhabdoviridae* group. The scale bar indicates the number of amino-acid substitutions per site. (**A**) Classification of viruses into different genera within the *Rhabdoviridae* family. The sequences identified in this study are surrounded in red. (**B**) Zoomed-in view of sequences assigned to Guadeloupe Culex rhabdovirus. The branch encircled in red represents unclassified rhabdoviruses. The sequences identified in this study are written in red. (**C**) Detailed view of the newly identified sequences in our study, tentatively named “Cambodia-Anopheles Rhabdoviridae-like”. These sequences are written in red. The branch encircled in red represents unclassified rhabdoviruses.

Phylogenetic analysis placed our sequences in a clade of unclassified *Rhabdoviridae* that mainly contains MAVs (Figure 7A,B).

Partial sequences were attributed to Culex pseudovishnui rhabdo-like virus and Merida virus, belonging to the genera *Ohlsrhavirus* and *Merhavirus* (Table 1), respectively. Sequences of the Culex pseudovishnui rhabdo-like virus were identified in *Cx. vishnui* group pools and showed a 96% amino-acid identity with the LC514056 strain from *Culex* mosquitoes identified in Japan. Sequences of Merida virus were detected in different *Culex* mosquito pools with a 99% amino-acid identity compared with the MH310083 strain discovered in *Cx. quinquefasciatus*.

All of the above-mentioned viruses have been referred to in the literature as MAVs because they have been identified only in mosquitoes. So far, no information of their transmissibility to vertebrates is available.

In addition, we detected a novel virus in *Anopheles* mosquito pools, tentatively named “Cambodia-Anopheles Rhabdoviridae virus”. It shared approximately 27% amino acid identity with the glycoprotein of the Ngaingan hapavirus (NGAV). Furthermore, our pipeline also indicated that the same virus exhibited 46% amino acid identity with the RdRp of Evro rhabdovirus (Table 1). NGAV belongs to the *Hapavirus* genus and has been isolated for the first time in 1970 from biting midges collected at the low-lying plains of the Mitchell River Aboriginal community, Gulf of Carpentaria, northern Queensland. Early serologic surveys have suggested that NGAV infects wallabies, kangaroos, and possibly cattle [48]. Evro rhabdovirus has only been identified in *Anopheles* mosquitoes. A phylogenetic analysis of the polymerase sequences placed this new virus in a distinct clade close to the group of “unclassified *Rhabdovirus*” that exclusively contains MAVs (Figure 7A,C).

#### 3.4.4. Orthomyxoviridae

The family *Orthomyxoviridae* comprises seven genera, *Isavirus*, *Thogotovirus*, and *Quaranjavirus* and the four types *of Influenza virus* (*Alpha*, *Beta*, *Delta, and Gamma*). The genera of Influenza virus contain viruses that cause influenza in birds and mammals, including humans. *Thogotoviruses* and *Quaranjaviruses* comprise arboviruses transmitted by ticks or mosquitoes.

Our results identified sequences assigned to Guadeloupe mosquito quaranja-like virus 1 (GMQLV1) and Wuhan Mosquito Virus 6 (WMV6), which belong to the *Quaranjavirus* genus (Table 1). Additionally, WMV6 viruses detected in *Cx. vishnui* group and those identified in *Cx. quinquefasciatus* mosquito species, shared more than 99% nucleotide identity.

The genomes of *Quaranjavirus* members usually contain six to seven segments [49]. Here, we identified four segments of GMQLV1 (PB1, PB2, PA, and NP) and five segments of WMV6 (PB1, PB2, PA, NP, and HA), respectively, in *Ae. aegypti* and *Culex* sp. pools. Only two quaranjaviruses have been recognized by the ICTV, the Johnston Atoll virus (JAV) and the Quaranfil virus (QRFV), known to be transmitted to birds by ticks [50]. QRFV is the only virus known to infect humans. It has been isolated from soft ticks and from the blood of children with mild febrile illness in Quaranfil, Egypt [51]. Numerous viruses have been included in the *Quaranjavirus* genus and have been designated as unclassified *Quaranjavirus* [52,53,54].

**Figure 8:**
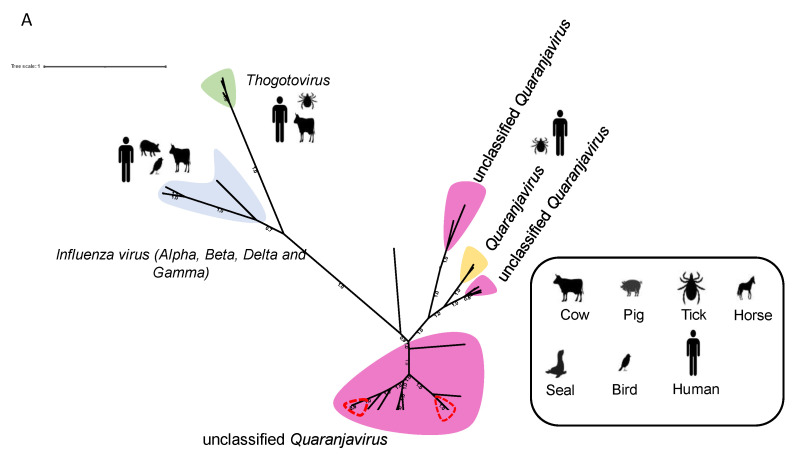

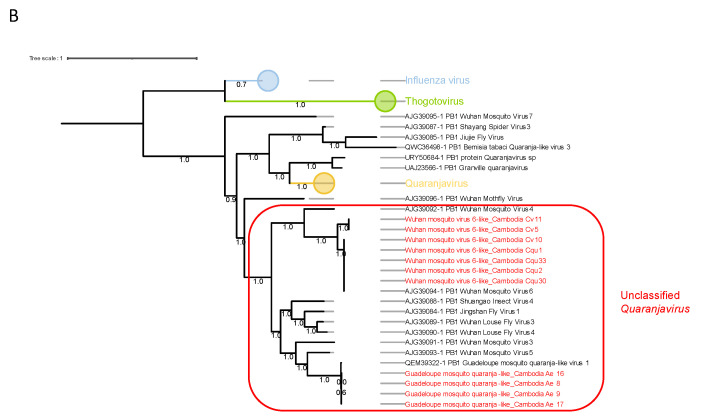
Phylogenetic analysis of *Orthomyxoviridae* viruses incorporated those identified in this study. The phylogenetic tree is constructed based on the PB1 amino acid sequence. (**A**) Classification of viruses into different genera within the *Orthomyxoviridae* family. The sequences identified in this study are surrounded in red. The labeled branches represent various known virus genera belonging to the *Orthomyxoviridae* family, as well as the sequences belonging to the unclassified *Quaranjavirus* group. The scale bar indicates the number of amino-acid substitutions per site. (**B**) Zoomed-in view of unclasified *Quaranjavirus* sequences. The branch encircled in red consists of viruses belonging to the unclassified *Quaranjavirus* group, including the viruses identified in our study. The sequences identified in this study are written in red.

The phylogenetic tree based on the PB1 segment of GMQLV1 and WMV6 and other *Orthomyxoviridae*-related viruses placed GMQLV1 and WMV6 in a clade of unclassified *Quaranjavirus* that mainly contains MAVs (Figure 8A,B).

#### 3.4.5. Birnaviridae

The *Birnaviridae* family includes seven genera and comprises viruses that infect a large diversity of hosts: *Aquabirnavirus*, *Avibirnavirus,* and *Blosnavirus* are genera known to infect vertebrates (excluding mammals), while the *Entomobirnavirus* and *Dronavirus* genera infect insects. In addition, the *Ronavirus* genus has been discovered from rotifers and the *Telnavirus* has been identified from molluscs. The genome of *Birnaviridae* viruses contains two segments, A and B encoding the polyprotein and the viral polymerase, respectively [55].

We identified viral sequences from *Cx. quinquefasciatus* pools that showed 37% amino-acid identity with the polyprotein segment of Port Bolivar virus and 51% amino-acid identity with the polymerase segment of Eridge virus, belonging to the *Entomobirnavirus* genus (Table 1). Port Bolivar has been isolated from a pool of *Ae. sollicitans* mosquitoes collected in East Texas, USA [56], and the Eridge virus was identified in Drosophila immigrans collected in the United Kingdom in 2011. We tentatively named the new virus “Cambodia Culex Birnaviridae virus”.

**Figure 9:**
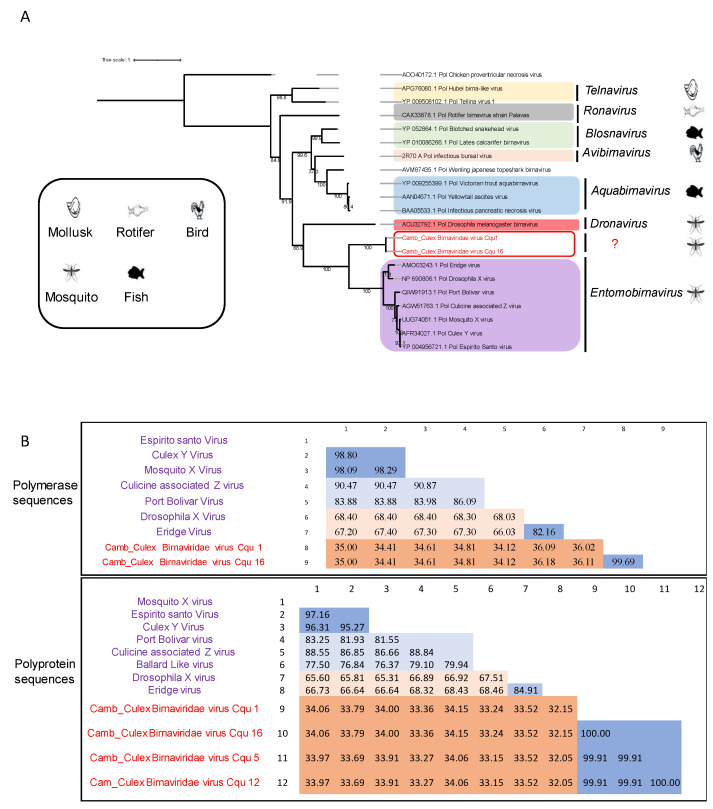
Phylogenetic analysis of *Birnaviridae* viruses incorporating those identified in this study. The phylogenetic tree was constructed based on the RdRp amino acid sequence. (**A**) Classification of viruses into different genera within the *Birnaviridae* family. The labeled branches represent various known virus genera belonging to the *Birnaviridae* family, as well as the newly identified sequences in this study. The sequences identified in this study are highlighted in red. The scale bar indicates the number of amino-acid substitutions per site. (**B**) Pairwise alignment of the polymerase and polyprotein segments between entomobirnaviruses and the new sequences identified in this study belonging to the *Birnaviridae* family.

We detected the complete sequence of the glycoprotein in four pools and the complete sequence of the polymerase in two pools. Phylogenetic analysis based on the polymerase protein revealed that the new viral sequences formed a distinct clade separate from other genera within the *Birnaviridae* family (Figure 9A). The closest clade to our sequences is the *Entomobirnavirus* genus.

To investigate the similarities between the new sequences and the entomobirnaviruses, we performed a pairwise comparison of the polymerase protein. The distance matrix showed an average amino acid identity of 35% between the novel sequences and the entomobirnaviruses for both segments (Figure 9B).

#### 3.4.6. Characterization of Some Unclassified Viruses

Because distinct characteristics or key information necessary for classification are missing, some viruses remain unclassified within existing taxonomic categories. We detected a significant number of taxonomically unclassified viruses in all mosquito species. In accordance with the results obtained for the classified viruses, we observed that the identified closest viral species varied among the mosquito genera. For example, sequences related to Humaita-Tubiacanga virus were detected exclusively in Aedes aegypti pools while sequences assigned to Hubei partiti-like virus 22, Hubei virga-like virus 2, and Broome luteo-like virus 1 were detected uniquely in Culex mosquito pools. It is worth noting that all the identified viruses in this group have previously been discovered solely in mosquitoes in earlier studies. These viruses have been referred to as MAVs (Table 1).

## 4. Discussion

Mosquitoes are known to be the principal vectors of arboviruses, which pose a major threat to human health. Numerous metagenomic analyses have been conducted on mosquitoes worldwide to characterize the viral communities carried by these vectors. In this study, we describe the diversity and evolution of viral communities associated with mosquitoes collected over one year in Kampong Thom Province, Cambodia.

A total of 26 viral taxa were identified, including viruses specific to vertebrates, invertebrates, plants, and fungi. Additionally, several viruses that remain unclassified by the ICTV were also identified.

We observed that while certain taxa of viruses are shared between different mosquito genera, some viral groups are restricted to specific mosquito genera. These findings align with previous studies, indicating that the composition of viral communities present in mosquitoes varies depending on the mosquito genus [57,58,59]. This supports the hypothesis that viral composition can be host-specific, implying that different mosquito species or genera may have unique viral associations. Several factors could contribute to host specificity. One of these factors can be the mosquito’s microbiota, which can interact with viruses, either directly or indirectly, impacting the abundance, replication, or transmissibility of the viruses [60]. Studies have reported variations in the composition and diversity of microbiota among different mosquito genera, leading to differences in the composition of the virome between mosquito genera [61,62].

Contrary to some previous studies that reported changes in virome composition according to the collection season [63,64], we did not observe such an association in Cambodian mosquitoes. This absence of difference can be explained by the fact that the presence of mosquito larval habitats harboring these viruses may not depend on seasonal precipitation and can persist throughout the year. These habitats can be permanent, such as lakes, rivers, and ponds, and provide continuous opportunities for virus transmission and the maintenance of mosquito populations [65]. It is important to consider these factors when studying the dynamics of virome composition in mosquitoes. Further research is needed to investigate the specific mechanisms that allow certain viruses to maintain a permanent presence in mosquito populations throughout the year.

We observed no differences in virome composition between mosquitoes captured inside households and those collected outside. This finding is not highly unexpected as it is conceivable that the same mosquitoes may move freely between indoor and outdoor environments.

To identify putative arboviruses responsible for mild infections in the human population, we focused on the taxa of viruses known to infect both vertebrates and invertebrates, and we characterized the viral species detected within these groups by performing a phylogenetic analysis. Despite the ongoing circulation of DENV and JEV in Cambodia, we did not detect these viruses in our samples. However, we identified one known arbovirus, CHIKV, in a pool of *Ae. aegypti* mosquitoes collected during the rainy season. The CHIKV strain identified in our study showed close genetic similarity (>99.9%) to the CHIKV strain identified in a serum sample from a patient in Cambodia in 2021. This finding demonstrates the effectiveness of our approach in detecting known arboviruses. However, it also highlights the challenges of detecting pathogenic arboviruses in field-caught mosquitoes, as reported in previous studies [66,67,68,69]. Similar to many prior studies, we chose to pool multiple mosquitoes before sequencing, which may have resulted in a decreased proportion of viral reads and reduced precision in characterizing mosquito virome profiles. However, a study that utilized a single mosquito for viral metagenomics did not observe a significant difference compared to using mosquito pools [58].

The challenges of detecting pathogenic arboviruses in field-caught mosquitoes may be attributed to several factors, including the low frequency of these pathogenic viruses within mosquito populations. Arboviruses can have a low prevalence in mosquito populations, making their detection more challenging compared with other more abundant viruses.

The most detected viruses were ISVs, including the cell fusing agent virus and Culex flavivirus, and MAVs, such as Guadeloupe Culex rhabdovirus and Wuhan mosquito virus 9. It is important to highlight that the characterized viral species in this study differ based on mosquito genus rather than species. Additionally, all sequences assigned to each viral species shared more than 99% nucleotide similarity, even when they were identified in different mosquito species and collected during different seasons. This suggests the presence of consistent viral species circulating among diverse mosquito populations throughout the year. This finding strengthens the earlier observation that virome composition remains unaffected by seasonal variations.

In the majority of metagenomics studies focusing on mosquito viromes, ISVs were found to be more abundant than arboviruses [20,70,71]. The abundance of ISVs within mosquitoes has been extensively studied to better understand their role and their potential impact on arbovirus transmission. Previous research on various *Culex* species revealed that mosquitoes infected with Culex Flavivirus (CxFV) showed reduced susceptibility to secondary infection with West Nile virus (WNV) compared with uninfected mosquitoes [72]. Another study demonstrated the ability of Palm Creek virus (PCV) to effectively reduce the replication of Kunjin virus (KUNV) and Murray Valley (MVEV) virus in C6/36 cells when co-infected with ISV [73]. However, a recent study found no significant effects of Palm Creek virus (ISV) infection on the vector competence of Zika and Chikungunya viruses in *Ae. aegypti* and *Ae. albopictus* mosquitoes [74]. These findings suggest that the impact of ISVs on arboviruses may vary depending on the specific arbovirus species, highlighting the need for cautious interpretation. However, it is crucial to acknowledge that our current understanding lacks conclusive evidence regarding the overall effects of ISV infection on other arthropod-borne pathogens. Further studies should be conducted to determine the underlying mechanisms by which ISVs interfere with the transmission of arboviruses and identify the host factors associated with their restriction of viruses to mosquito hosts.

The increasing number of metagenomics studies focusing on the virome associated with mosquitoes has led to the discovery of many MAVs in recent years. However, limited information is available regarding their transmissibility to vertebrates and their specific hosts. Given their abundance and persistence, it is important to study the potential role of MAVs in arbovirus transmission and their interactions with specific hosts. Some MAVs, such as Hubei partiti-like virus 22 and Hubei virga-like virus 22, were identified in our study and belong to the “unclassified virus” group. This group comprises viruses for which crucial information, such as their classification, evolutionary history, genetic diversity, and ecological characteristics, is currently unknown. Further investigations should be conducted to classify the viruses within this group.

Interestingly, we identified three novel viruses for the first time. The first one appears to be the virus identified in *Cx. brevipalpis*, which shares 71% amino acid identity with a Culex flavivirus. However, phylogenetic analyses have placed it within the MAVs clade in the group of unclassified *Flavivirus*, and it is not very distant from Culex flaviviruses. The second virus, Cambodia-Anopheles Rhabdoviridae virus, was classified within a new clade among the *Rhabdoviridae* family. This virus is closely related to a clade exclusively containing MAVs, indicating that it could represent a novel group of MAVs within the *Rhabdoviridae* family. The third virus, named Cambodia Culex Birnaviridae virus, was placed in a distinct clade, significantly distant from the various genera within the *Birnaviridae* family. The closest clade to our sequences was identified as the *Entomobirnavirus* genus. Conducting a pairwise comparison of the polymerase protein between entomobirnaviruses and Cambodia_Culex Birnaviridae-like virus revealed an average amino acid identity of 35%. Based on the demarcation criteria set by the ICTV for the *Birnaviridae* family, we propose that the Cambodia Culex Birnaviridae-like virus belongs to a novel genus within the *Birnaviridae* family. To further characterize these new viruses, additional studies are needed to determine their ability to infect vertebrates, such as molecular surveillance of patients with an unknown fever etiology or serological surveys of human populations continuously exposed to mosquito bites.

## 5. Conclusions

This study shows the diversity of viral communities, encompassing both classified and unclassified viruses. It also reinforces the hypothesis that viral composition can be host-specific, particularly within the mosquito genus, which harbors unique viral associations. Insect-specific viruses (ISVs) and mosquito-associated viruses (MAVs) were found to be more abundant than arboviruses. The study also highlights the presence of novel and unclassified viruses, underscoring the need for further research to determine their infectivity and potential impact on vertebrates.

## Figures and Tables

**Table 1:** Compilation of referenced the Last Common Ancestors (LCAs) and their associated traits. The LCAs represented here are those identified by the Microseek pipeline.

Mosquito Species Harboring Viruses	LCA (Last Common Ancestor)	Genbank Accession Number of the LCA	Family	Genus	Primary Known Host	Maximum % aa Identity	Complete Genome/Complete CDS	Positive Libraries/Tested Libraries (%)
*Aedes aegypti*	Chikungunya virus	OL999095.1	*Togaviridae*	*Alphavirus*	Human/vertebrate/Invertebrate	100	No	1/19 (5)
*Aedes aegypti*	Cell fusing agent virus	LR694078.1	*Flaviviridae*	*Flavivirus*	*Aedes*	100	Yes	17/19 (89)
*Culex vishnui.g*	Culex flavivirus	HQ678513.1	*Flaviviridae*	*Flavivirus*	*Culex*	99	Yes	5/24 (20)
*Anopheles vagus*	Culex flavivirus	BBQ04787	*Flaviviridae*	*Flavivirus*	*Culex*	71	No	2/10 (20)
*Culex quinquefasciatus*	Culex flavivirus	HQ678513.1	*Flaviviridae*	*Flavivirus*	*Culex*	99	Yes	35/35 (100)
*Culex brevipalpis*	Culex flavivirus	HQ678513.1	*Flaviviridae*	*Flavivirus*	*Culex*	98	Yes	4/5 (80)
*Culex brevipalpis*	Culex flavivirus	MN318426.1	*Flaviviridae*	*Flavivirus*	*Culex*	71	Yes	1/5 (20)
*Culex gelidus*	Quang binh Virus	NC_012671.1	*Flaviviridae*	*Flavivirus*	*Culex*	99	Yes	2/3 (67)
*Culex vishnui.g*	Guadeloupe Culex rhabdovirus	MN013393.1	*Rhabdoviridae*	Unclassified	Mosquitoes	100	Yes	8/24 (33)
*Culex quinquefasciatus*	Guadeloupe Culex rhabdovirus	MN013393.1	*Rhabdoviridae*	Unclassified	Mosquitoes	100	Yes	35/35 (100)
*Culex brevipalpis*	Guadeloupe Culex rhabdovirus	MN013393.1	*Rhabdoviridae*	Unclassified	Mosquitoes	100	Yes	1/5 (20)
*Culex tritaeniorhynchus*	Wuhan Mosquito virus 9	YP_009305109.1	*Rhabdoviridae*	Unclassified	*Culex*	100	No	1/2 (50)
*Anopheles vagus*	Ngaingan Hapavirus (Glycoprotein)	YP_003518289.1	*Rhabdoviridae*	*Hapavirus*	*Anopheles*	27	Yes	8/10 (80)
	Evro rhabdovirus (RdRp)	QRD99862.1	*Rhabdoviridae*	Unclassified	*Anopheles*	46	Yes	8/10 (80)
*Anopheles indefinitus*	Ngaingan Hapavirus	YP_003518289.1	*Rhabdoviridae*	*Hapavirus*	*Anopheles*	27	No	2/3 (67)
*Culex vishnui.g*	Merida virus	MH310083	*Rhabdoviridae*	*Merhavirus*	*Culex*	99	No	2/24 (8)
*Culex quinquefasciatus*	Merida virus	MH310083	*Rhabdoviridae*	*Merhavirus*	*Culex*	99	No	9/35 (26)
*Culex brevipalpis*	Merida virus	MH310083	*Rhabdoviridae*	*Merhavirus*	*Culex*	99	No	1/5 (20)
*Culex vishnui.g*	Culex pseudovishnui rhabdo-like	LC514056.1	*Rhabdoviridae*	*Ohlsrhavirus*	*Culex*	96	No	12/24 (50)
*Aedes aegypti*	Guadeloupe mosquito quaranja-like virus 1 (RdRp)	QRW42587.1	*Orthomyxoviridae*	*Quaranjavirus*	*Aedes*	99	Yes	16/19 (84)
*Culex vishnui.g*	Wuhan Mosquito Virus 6 (RdRp)	QRW42421.1	*Orthomyxoviridae*	*Quaranjavirus*	*Culex*	100	Yes	14/24 (58)
*Culex quinquefasciatus*	Wuhan Mosquito Virus 6 (RdRp)	QTW97780.1	*Orthomyxoviridae*	*Quaranjavirus*	*Culex*	100	Yes	35/35 (100)
*Culex quinquefasciatus*	Port Bolivar virus (Polyprotein)	QIW91912.1	*Birnaviridae*	*Entomobirnavirus*	*Aedes*	51	Yes	4/35 (11)
	Eridge virus (RdRp)	AMO03243.1	*Birnaviridae*	*Entomobirnavirus*	*Aedes*	35	Yes	2/35 (6)
*Aedes aegypti*	*Humaita-Tubiacanga*	OQ305261.1	Unclassified	Unclassified	Mosquitoes	100	Yes	14/19 (74)
*Culex quinquefasciatus*	*Hubei partiti-like virus 22*	MW452285.1	Unclassified	Unclassified	*Culex*	100	Yes	24/35 (68)
*Culex tritaeniorhynchus*	*Hubei partiti-like virus 22*	MW452285.1	Unclassified	Unclassified	*Culex*	100	No	1/2 (50)
*Culex quinquefasciatus*	*Hubei virga-like virus 2*	MW452285.1	Unclassified	Unclassified	*Culex*	99	Yes	30/35 (86)
*Culex vishnui.g*	*Hubei virga-like virus 2*	MW452285.1	Unclassified	Unclassified	*Culex*	99	Yes	1/24 (1)
*Culex vishnui.g*	*Broome luteo-like virus 1*	MT498823.1	Unclassified	Unclassified	*Culex annulirostris*	91	Yes	17/24 (71)
*Culex tritaeniorhynchus*	*Broome luteo-like virus 1*	MT498823.1	Unclassified	Unclassified	*Culex annulirostris*	85	Yes	1/2 (50)

## Data Availability

The raw data of classified viruses, for which we obtained complete genomes or, in the case of segmented viruses, all the segments in full, were deposited into the NCBI/SRA database and are available under the Bioproject number PRJNA1008583. The sequences of these viruses were deposited in GenBank under accession numbers OR479681; OR479682; OR479683; OR479684; OR479685; OR479686; OR479687; OR479688; OR479689; OR479690; OR479691; OR479692; OR479693; OR479694; OR479695; OR479696; OR479697; OR479698; OR479699; OR479700; OR479701.

## Supplementary Materials

The following supporting information can be downloaded at: https://www.mdpi.com/article/10.3390/v15091831/s1. Figure S1: Map locating the municipalities where mosquitoes were collected. Figure S2: Distribution of mosquito species collected during the dry season and the rainy season. Figure S3: Histogram visualizing the shared and non-shared taxa among different mosquito genera. Figure S4: Principal Coordinates Analysis (PCoA) to assess the influence of season and location on the virome composition. Table S1: Information about mosquito pooling pool name, mosquito species, season, and location. Table S2: Information about raw reads obtained et viral abundances. Table S3: Fold Change and adjusted *p*-values for all viral groups resulted in differential analysis.
