# Supplementary material for: Longitudinal Study of Viral Diversity Associated with Mosquito Species Circulating in Cambodia
Source: Viruses. 2023 Aug 29;15(9):1831. doi: 10.3390/v15091831 (PMC10535147; doi:10.3390/v15091831)
Supplement: Supplementary file 1 [file viruses-15-01831-s001.zip › Fig.S4 PCoaA covariates.pdf]

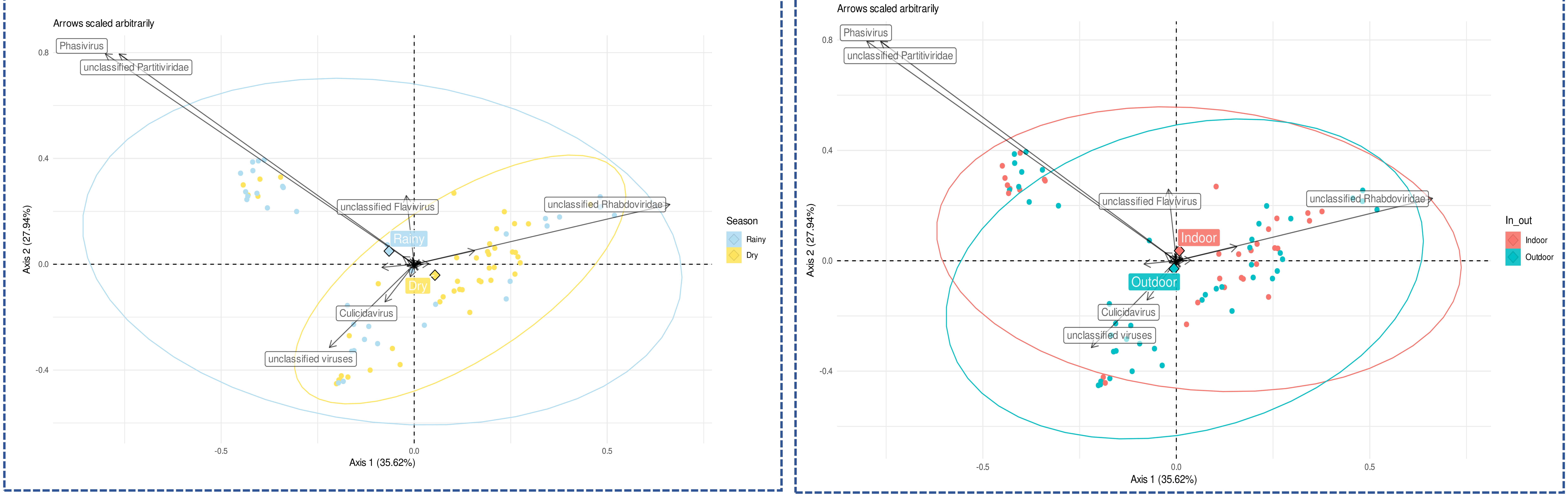

(a) (b)

Figure S4: Principal Coordinates Analysis (PCoA) to assess the influence of season and location on the virome composition. The first two axes accounted for 63% of the variability in the data. (a) PCoA reveals differences in viral composition across seasons. (b) PCoA demonstrates differences in viral composition between indoor and outdoor locations.
