# Supplementary figures and images for: Longitudinal Study of Viral Diversity Associated with Mosquito Species Circulating in Cambodia
Source: Viruses. 2023 Aug 29;15(9):1831. doi: 10.3390/v15091831 (PMC10535147; doi:10.3390/v15091831)

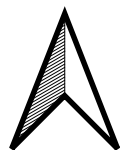

THAILAND

LAO PDR

VIETNAM

Kampong Thom

0 75 150 km

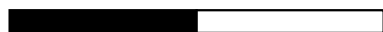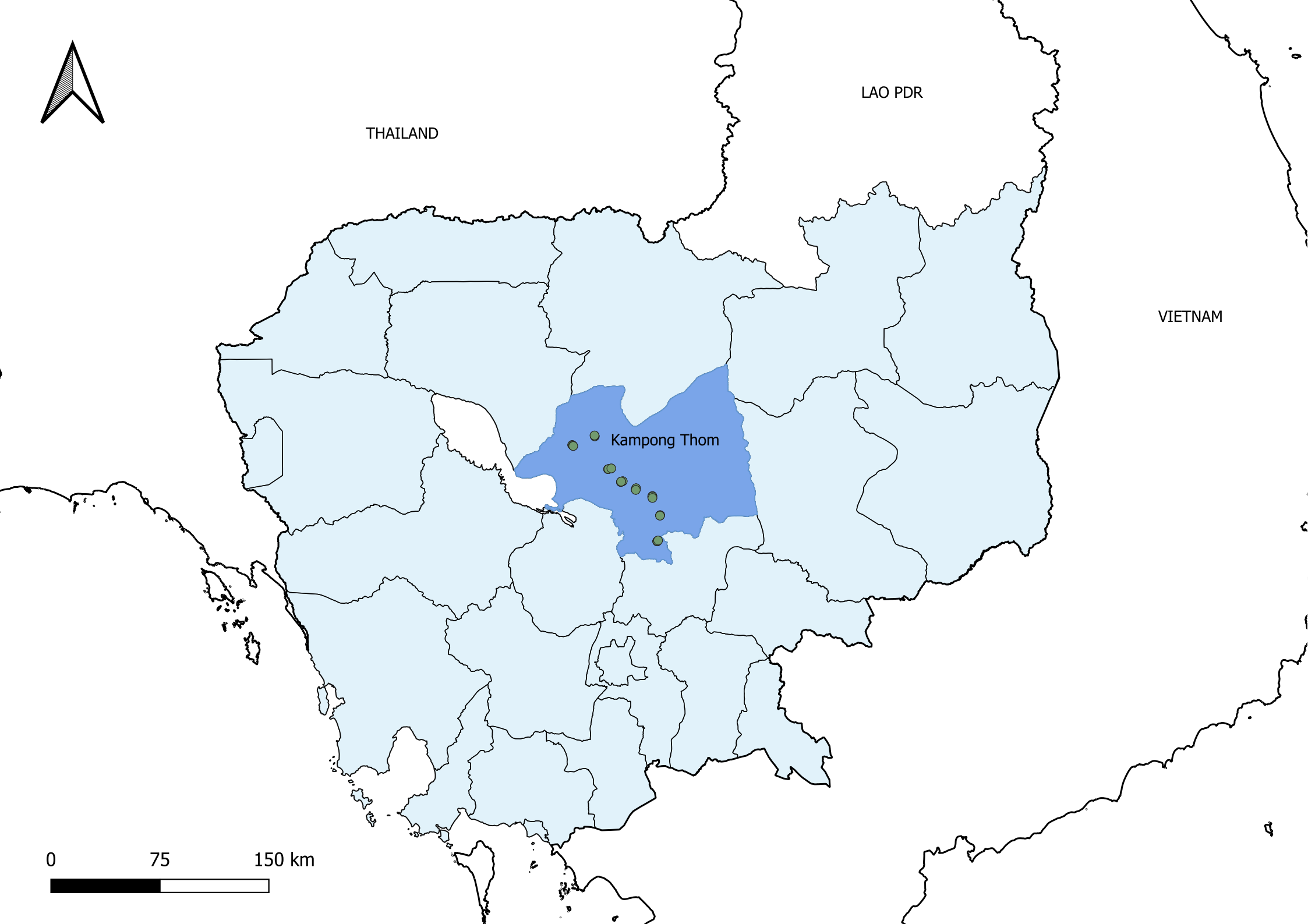

Supplement: Supplementary file 1 [file viruses-15-01831-s001.zip › Fig.S1 Map locating the municipalities where mosquitoes were collected.pdf]

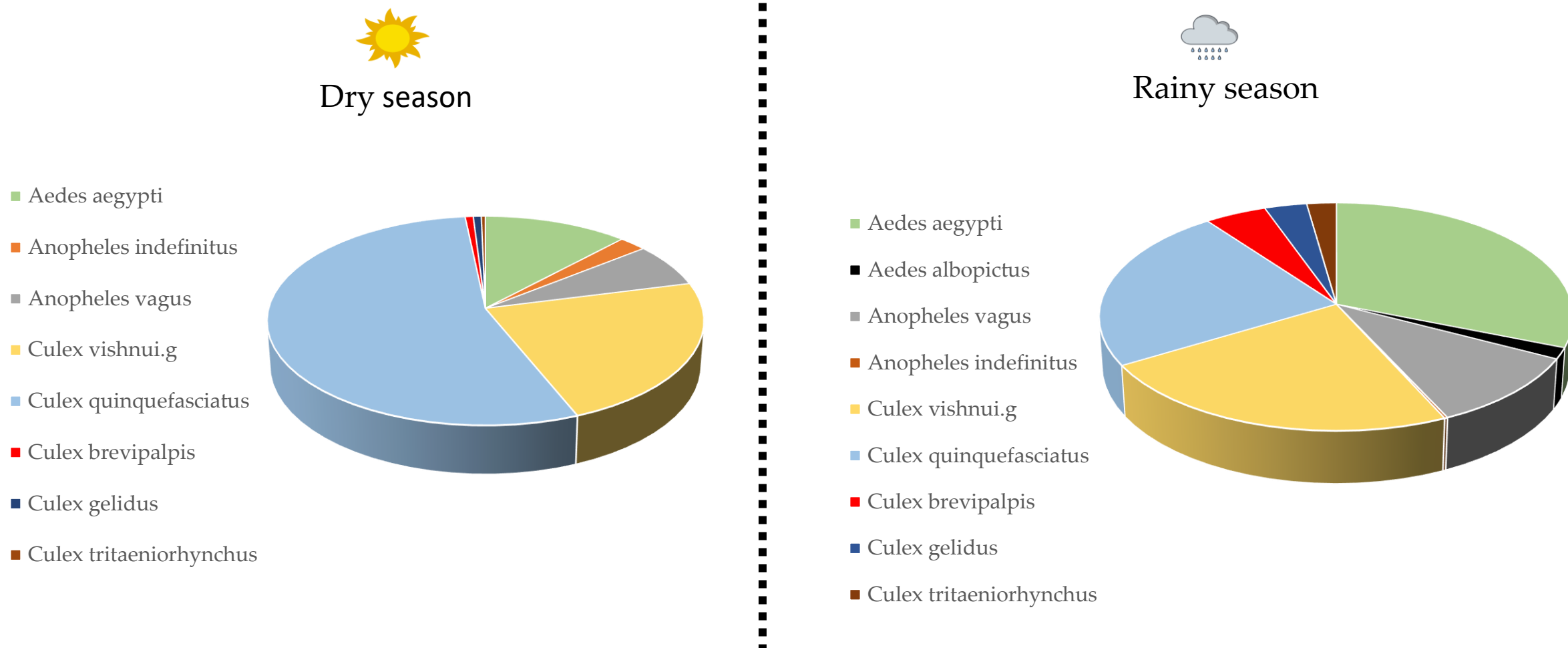

Fig S2: Distribution of mosquito species collected during the dry season and the rainy season

Supplement: Supplementary file 1 [file viruses-15-01831-s001.zip › Fig S2 Distribution of mosquito species collected during the dry season and the rainy season.pdf]

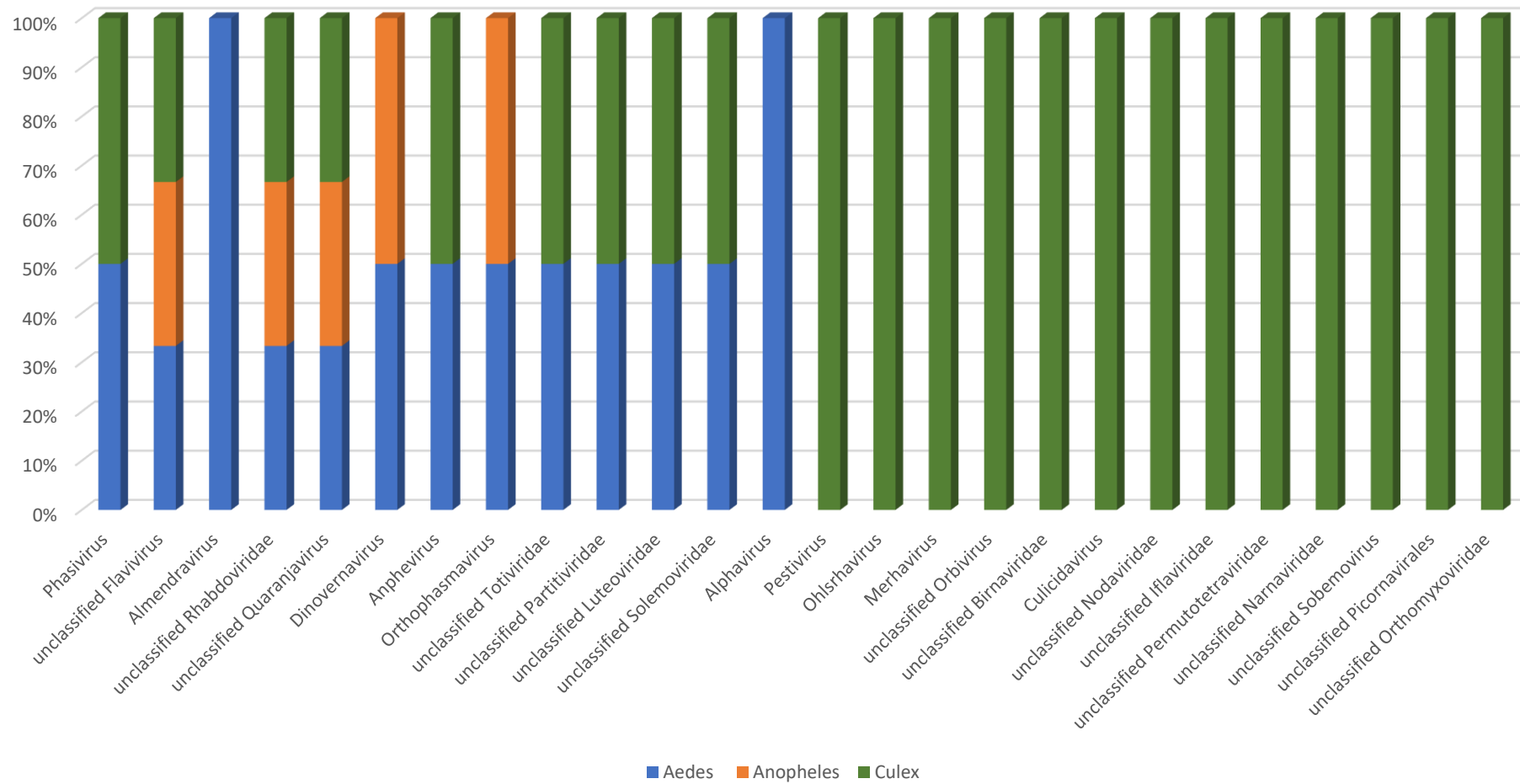

Fig S3: Histogram visualizing the shared and non-shared taxa among different mosquito genera

Supplement: Supplementary file 1 [file viruses-15-01831-s001.zip › Fig S3 Histogram visualizing the shared and non-shared taxa among different mosquito genera.pdf]
